# Oral bovine colostrum supplementation does not increase circulating insulin-like growth factor-1 concentration in healthy adults: results from short- and long-term administration studies

**DOI:** 10.1007/s00394-019-02004-6

**Published:** 2019-05-23

**Authors:** Glen Davison, Arwel W. Jones, Tania Marchbank, Raymond J. Playford

**Affiliations:** 1 School of Sport and Exercise Sciences, University of Kent at Medway, Chatham, UK; 2 Lincoln Institute for Health, University of Lincoln, Lincoln, UK; 3 Faculty of Medicine & Dentistry, University of Plymouth, Plymouth, UK; 4 Centre for Immunobiology, Blizard Institute, Barts and The London School of Medicine, Queen Mary University of London, London, UK; 5 Peninsular Medical School, University of Plymouth, Plymouth, UK

**Keywords:** Nutraceutical, Sports nutrition, Food supplement, Prostate cancer

## Abstract

**Purpose:**

Bovine colostrum is available in health food shops and as a sports food supplement and is rich in antibodies and growth factors including IGF-1. World Anti-Doping Agency advises athletes against taking colostrum for fear of causing increased plasma IGF-1. There are also concerns that colostrum may theoretically stimulate malignancy in organs which express IGF-1 receptors. We, therefore, determined changes in plasma IGF-1 levels in subjects taking colostrum or placebo for 1 day, 4 weeks, and 12 weeks.

**Methods:**

Plasma IGF1 levels were determined in healthy males (*n* = 16) who ingested 40 g bovine colostrum or placebo along with undertaking moderate exercise for total period of 4.5 h. Two further studies followed changes in IGF1 using double-blind, parallel group, placebo-controlled, randomized trials of colostrum or placebo (*N* = 10 per arm, 20 g/day for 4 weeks and *N* = 25 colostrum, *N* = 29 placebo arm 20 g/day for 12 weeks).

**Results:**

Baseline IGF1 levels 130 ± 36 ng/ml. 4.5 h protocol showed no effect of colostrum on plasma IGF1 (ANOVA, treatment group: *p* = 0.400, group × time: *p* = 0.498, time *p* = 0.602). Similarly, no effect of colostrum ingestion was seen following 4 week (ANOVA, group: *p* = 0.584, group × time interaction: *p* = 0.083, time *p* = 0.243) or 12 week (ANOVA, group: *p* = 0.400, group × time interaction: *p* = 0.498, time *p* = 0.602) protocol.

**Conclusions:**

Ingestion of standard recommended doses of colostrum does not increase IGF-1 levels in healthy adults, providing additional support for the safety profile of colostrum ingestion.

## Introduction

Gastrointestinal symptoms including cramps, diarrhea, nausea, and bleeding are commonly reported by long-distance runners and athletes undertaking extreme endurance competitions and are likely to be due to a combination of reduced splanchnic blood flow, hormonal changes, altered gut permeability, and increased body temperature [1–4]. These athletes are also susceptible to increased incidence of symptoms of upper respiratory tract infections, especially during periods of strenuous exercise training and immediately after competition [5, 6].

Pharmacological options to reduce these problems are limited, particularly in competitive athletics, and there is, therefore, great interest in the use of natural products. One such product, that is already commercially available, is bovine colostrum. Colostrum is the first milk produced after birth and is particularly rich in immunoglobulins, antimicrobial peptides (e.g., lactoferrin, lactoperoxidase), and other bioactive molecules including growth factors such as transforming growth factor-beta and IGF-1 [7]. As an example, IGF1 levels in bovine colostrum are approximately fivefold higher than mature milk [8]. In combination with the milk that is subsequently produced, colostrum is important for the nutrition, growth, and development of the new-born infant and contributes to the immunological defense of the neonate and in eliminating infection and stimulating growth of the neonatal gastrointestinal tract [9]. In adults, randomized clinical trials have shown beneficial effects of oral colostrum supplementation in reducing NSAID-induced and exercise-induced hyperpermeability [10, 11] and reducing the frequency of upper respiratory symptoms of athletes in training [12].

Colostrum is freely available in health food stores and via the internet and is taken by normal subjects to maintain gut integrity, patients with gut-related injuries and athletes to enhance their ability to train successfully. However, the world anti-doping agency (WADA) advises athletes against taking colostrum for fear of causing a rise in levels of IGF-1 in the circulation with resulting doping penalties (https://www.wada-ama.org). In addition, concerns have been raised that if circulatory IGF-1 is raised in response to colostrum supplementation, prolonged administration of colostrum might stimulate malignancy in distant organs such as prostate cancer which is known to commonly express IGF-1 receptors [13].

To address these potential concerns, we now present studies examining the relationship between colostrum ingestion and circulatory levels of IGF-1. These studies comprised: (1) an analyses of changes in plasma IGF-1 levels following short term administration of colostrum; (2) an analyses of changes in IGF-I in stored plasma samples that were collected during two placebo-controlled randomized trials of longer term bovine colostrum supplementation (1 and 3 months, respectively) on immune health that were led by the authors of the present study [14, 15].

## Materials and methods

### Subject selection, collection of blood samples, IGF-1 assay and test products

All participants were recreationally active males, non-allergic to dairy products and reported no symptoms of infection or taking any medication or dietary supplements 4 weeks prior to commencement of studies. For all studies, blood samples were collected into a vacutainer (Becton–Dickinson, Oxford, UK) containing EDTA, and tubes centrifuged at 1500*g* for 10 min at 4 °C. Plasma was then isolated and stored at − 80 °C until analysis. On day of assay, plasma samples underwent pre-treatment to release IGF-1 from binding proteins and IGF-1 concentration then quantified in duplicate using a commercial enzyme-linked immunosorbent assay (ELISA, R&D Systems, Abingdon, UK) according to manufacturer’s instruction.

For all three studies, effects of powdered bovine colostrum, (Neovite UK, London, UK) was compared against an isoenergetic/isomacronutrient placebo (comprising skimmed milk powder and milk protein concentrate (Marvel, Premier Foods, Thame, UK and My Protein Ltd, Cheshire, UK).

### Study 1: effect of short-term bovine colostrum supplementation on plasma IGF-1 in subjects undertaking moderate exercise

Design: a double-blind, placebo-controlled, counterbalanced, randomized crossover protocol.

Participants: sixteen male subjects (age 25 ± 6 years; height 178 ± 6 cm; BM 75.7 ± 7.5 kg).

**Fig. 1:**
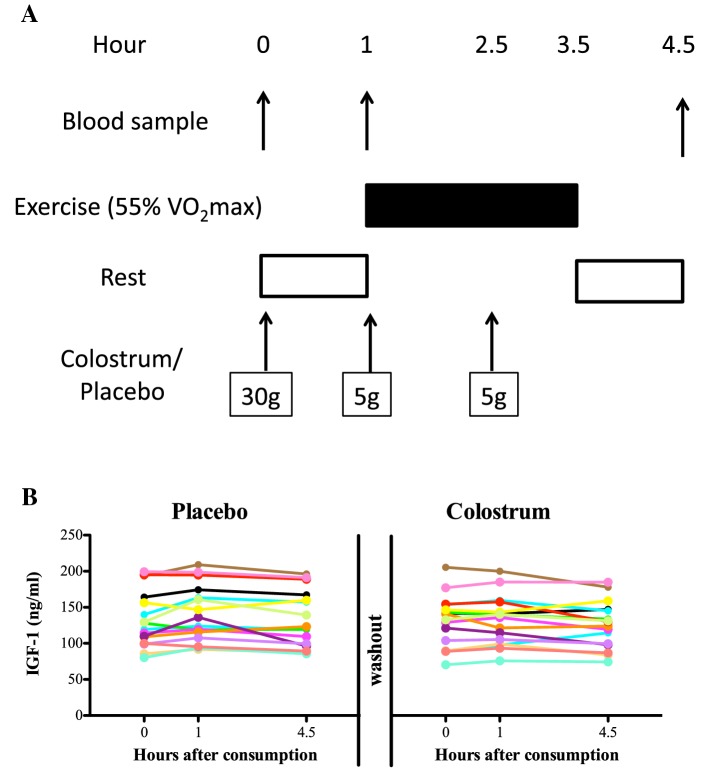
Effect of short -term bovine colostrum or placebo supplementation (40 g in total) on plasma IGF-1 in subjects undertaking moderate exercise using a randomized crossover design. **a** Study protocol. **b** Plasma IGF-1 levels found at the various stages of the study. Individual results are shown. No statistically significant differences between the two arms were seen

Protocol: each subject underwent two identical experimental arms, in randomized order, separated by 7 days (Fig. 1a). During the 48 h preceding each arm, participants abstained from heavy exercise and alcohol and recorded their food intake during the 24 h before the first experimental arm. To standardize their nutritional status, subjects followed the same food intake during the 24 h prior to second experimental arm.

Subjects reported to the laboratory after an overnight fast of at least 10 h. Upon arrival, participants remained seated for 10 min prior to collection of a resting baseline (0 h) blood sample. Participants then consumed either 30 g of bovine colostrum or the isoenergetic/isomacronutrient placebo (both made up in 300 ml water). Participants undertook restful activities (e.g., reading) before collection of second blood sample (+ 1 h sample) and then ingested a further 5 g of colostrum or placebo mixed in 50 mL water prior to starting 2.5 h of cycling at 15% Δ (~ 55–60% $V{\text{O}}_{2\hbox{max} }$). A final dose (5 g) of colostrum or placebo was given 1.25 h into the exercise regimen (+ 2.25 h sample) to all participants. Participants were permitted diluted cordial (four volumes of water to 1 volume of sugar-free cordial at 2 mL/kg of BM) every 15 min during the first arm with the pattern of intake and total volume consumed being replicated in the second arm. One hour after finishing the cycling a third and final blood sample (+ 4.5 h) was taken.

Statistical analyses were performed using the statistical programmed SPSS (v24.0; IBM Corp., Armonk, NY., USA) with statistical significance accepted at *p* < 0.05. Data were checked for normal distribution using the Shapiro–Wilk test. A two-factor repeated measures ANOVA (group × time) was then carried out.

### Studies 2 an 3: effect of long-term bovine colostrum supplementation (4 and 12 weeks) on plasma IGF-1 levels in healthy subjects

Summary of overall design: effects of colostrum administration on IGF-1 levels were determined using stored plasma samples collected from two double-blind, parallel group, placebo-controlled, randomized trials. In these trials, colostrum had been administered for 4 and 12 weeks with the primary aim of examining rate of respiratory infection, mucosal immunity and blood neutrophil responses [14, 15].

#### Study 2

Twenty (10 in each arm) individuals aged 28 ± 8 years; body mass 79 ± 7 kg participated in the study consisting of daily supplementation of 20 g of colostrum or placebo for 4 weeks. Resting blood samples were collected following an overnight fast at the beginning and end of the 4-week period [14].

#### Study 3

Fifty-seven subjects were randomized to receive daily supplementation of 20 g of colostrum or placebo (10 g in the morning and in the evening before relevant meal) for 12 weeks. Four subjects had protocol violation (failure to take supplementation for full period) and were excluded. Those that completed the full protocol comprised 25 individuals in colostrum arm (age: 30.5 ± 13.8 years, height: 179.9 ± 6.4 cm, body mass: 77.2 ± 8.9 kg) and 28 subjects (age: 31.5 ± 13.2 years, height 178.4 ± 6.6 cm, body mass 74.5 ± 8.7 kg) in placebo arm. Resting blood samples following an overnight fast were collected at the beginning and end of the 12-week period [15].

#### Statistical analyses

Statistical analyses were performed using SPSS and data were checked for normal distribution using the Shapiro–Wilk test. A two-factor mixed model ANOVA (group × time) was used to determine if the effect of time was different between bovine colostrum (4-week or 12-week supplementation) and placebo groups.

## Results

### Study 1: effect of short-term bovine colostrum supplementation on plasma IGF-1 in subjects undertaking moderate exercise

Results for each individual are shown in Fig. 1b. A two-factor repeated measures ANOVA (group: *p* = 0.133, group × time interaction: *p* = 0.166, time *p* = 0.013) revealed a slight non-significant change over time, due to a slight rise in both arms at the 1-h time period, but not any significant differences in IGF-1 between a total dose of 40 g of bovine colostrum (*n* = 16, baseline: 130 ± 36 ng/ml, + 1 h: 132 ± 34 ng/ml, + 4.5 h: 126 ± 32 ng/ml) or placebo (*n* = 16, baseline: 133 ± 39 ng/ml, + 1 h: 141 ± 39 ng/ml, + 4.5 h: 133 ± 39 ng/ml).

### Studies 2 and 3: effect of long-term bovine colostrum supplementation (4 and 12 weeks) on plasma IGF-1 levels in healthy subjects

#### Study 2: 4-week administration

**Fig. 2:**
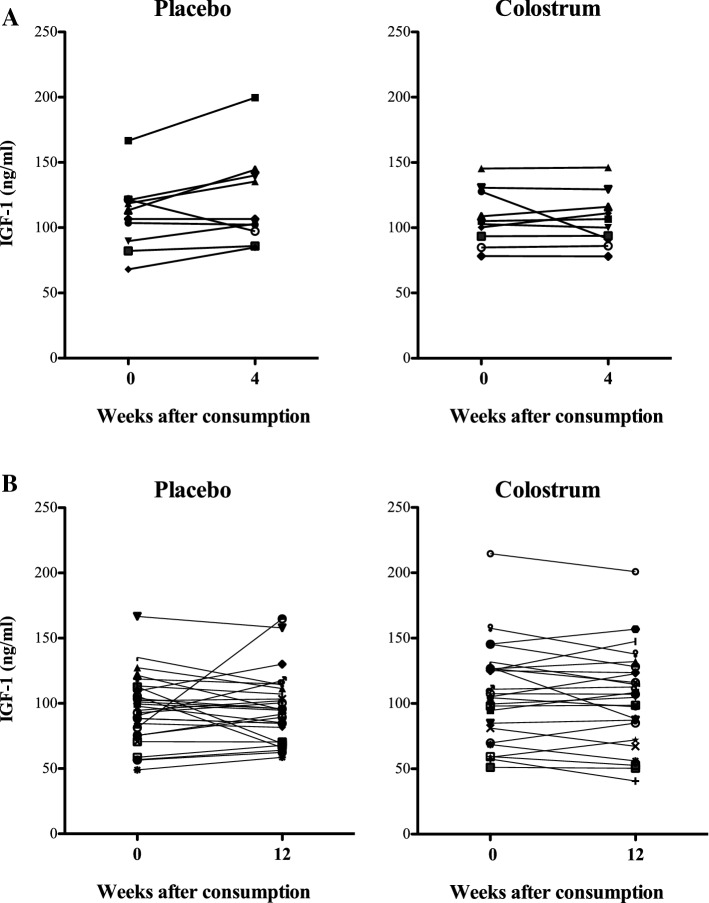
Effect of long-term bovine colostrum or placebo supplementation on IGF-1 levels using double-blind, parallel group, placebo-controlled, randomised protocols. **a** IGF-1 levels in subjects receiving 20 g per day for 4 weeks **b** IGF-1 levels in subjects receiving 20 g per day for 12 weeks. Individual results from each subject are shown. No statistically significant differences between placebo and colostrum were seen

Compared to placebo, 4 weeks of bovine colostrum (20 g per day) revealed no differences in circulating IGF-I between placebo and bovine colostrum (two-way mixed ANOVA, group: *p* = 0.584, group × time interaction: *p* = 0.083, time *p* = 0.243) (Fig. 2a).

#### Study 3: 12-week administration

Compared to placebo, 12 weeks of bovine colostrum (20 g per day) supplementation did not lead to changes in circulating IGF-I (two-factor mixed ANOVA, group: *p* = 0.400, group × time interaction: *p* = 0.498, time *p* = 0.602) (Fig. 2b).

## Discussion

We showed that oral consumption of bovine colostrum (20-40 g/day), either acutely (1–4.5 h post-ingestion) or chronically (4–12 weeks) in healthy adult subjects has no influence on systemic concentrations of IGF-I.

Growth factors, whether produced by purification or using recombinant technology, are increasingly being used for a variety of clinical conditions. Examples include recombinant human insulin for the treatment of diabetes, erythropoietin for renal failure‐induced anemia and interferon for viral hepatitis. The use of such factors for ‘hollow organ’ gastrointestinal conditions is at a more preliminary stage although systemically administered glucagon-like peptide-2 (GLP-2) for the treatment of short bowel syndrome is now a clinically proven, albeit very expensive treatment (estimate $200,000 per quality adjusted life year). In addition to cost, there are concerns that systemic growth factor administration may have unwanted side effects such as tumor progression as many tumors express several growth factor receptors and may also cause other unwanted side effects such as fluid retention.

In parallel with studies of recombinant peptides for gut conditions, there have been several clinical trials examining the potential value of bovine colostrum for gastrointestinal problems. Use of colostrum rather than a single recombinant peptide has the advantages of maintaining potential synergistic activity between the various growth factors within colostrum and that its formulation naturally protects its growth factor activity to reach more distal regions of the gut in an intact biologically active form [16]. These trials suggest that colostrum has clinical value for NSAID-induced gut injury [9], chemotherapy induced mucositis [17] and inflammatory bowel disease [18]. In addition, colostrum may have value for reducing symptoms of infective diarrhea [19], possibly acting through its specific (antibodies) and general antimicrobial constituents such as lactoferrin. Colostrum may also have value as a sports nutritional supplement to reduce the incidence of upper respiratory tract infections during training [15] and to reduce the increased gut permeability that occurs during heavy exercise [20]. Although there is currently insufficient evidence to conclusively establish the value of bovine colostrum to enhance performance, it is being used increasingly by track and field athletes. Of the studies that have been performed, its major value may be in the context of athletes undertaking high-intensity training and in aiding recovery [21].

In addition to its pharmacological advantages, there is currently a demand from the general public for more ‘natural’ types of products, which are usually considered as ‘alternative therapy’, but which can possess potent biological activity. Products such as these are often termed nutraceuticals (from nutrition and pharmaceuticals) [22].

Although there seems to be many advantages of using colostrum at the sports nutrition-functional food interface, two main concerns have been raised, especially if taken for prolonged periods. The first relates to use as a supplement in competitive sports training; The colostrum products used in these studies have been certified by a company (HFL Sports Science UK, Fordham, Cambridgeshire) that has ISO 17025 accreditation for testing sports supplements to control contamination with banned substances to ensure compliance with WADA and has been shown to not contain any banned substances. Nevertheless, because colostrum contains IGF-I, WADA advises athletes against taking colostrum for fear of causing a rise in the levels of IGF-I in the circulation with resulting doping penalties.

Although we are not aware of any clinical data supporting this idea, concerns have also been raised that colostrum might increase the risk of prostate cancer, as prostate cancer cells often express IGF-I receptors and respond to IGF-I administration with enhanced cell proliferation and glucose consumption. However, the pathophysiological situation is more complicated, as administration of IGF-I to noncancerous prostate cells increases basal to luminal differentiation [13].

It is for these reasons that there is much interest in whether circulatory IGF-I increases in subjects taking colostrum. Our initial study attempted to reproduce a standard scenario of a person taking standard doses of colostrum in combination with undertaking moderate exercise, as occurs during sports training. We found no statistically significant differences in plasma IGF-I levels in response to colostrum versus placebo. The second study examined moderate doses of colostrum (10 g twice a day for 4 weeks) reproducing an individual who decides to take a short course of colostrum supplementation and again found no rise in IGF-I levels in either placebo or colostrum group. The third study examined the same 20 g/day dose of colostrum for a prolonged period (12 weeks) and again, no rise was seen.

Our findings support and extend previous work by Coombes et al. who examined changes in plasma IGF-1 in response to 20–60 g per day of colostrum for 8 weeks and found no increase [7]. Similar negative results have been reported by others following ingestion of 20 g or 60 g/day of colostrum or whey, also for 8 weeks [23–25]. In contrast, Mero et al. reported that colostrum ingestion does increase IGF-I levels [26] although there were methodological and interpretation issues as the only significant effect seen was comparing the change in IGF-1 levels from baseline, due to the placebo arm falling by 18 ng/ml and the colostrum-treated group increasing by 28 ng/ml) [26]. These changes, therefore, seem to reflect physiological variability of IGF-I levels rather than a change due to colostrum ingestion, a conclusion supported by our results from our study 1. In addition, the Mero study [26] used glucose as the placebo rather than a protein containing control as used by other groups. This is important in interpreting their results, as higher protein intake is associated with increased endogenous IGF production [27].

Additional evidence against ingested IGF- I reaching the circulation intact comes from studies examining the effect of orally administered ^125^I labeled IGF-I on blood IGF-I [28]. These showed that virtually all the ^125^I present in the bloodstream was in a digested fragmentary inactive form. This suggests that any change seen in IGF-I levels would be more likely to be due to changes in intrinsic production rather than due to absorption of colostrum derived IGF-I.

In summary, we conclude that ingestion of standard doses of colostrum (20–40 g/day) in healthy adult subjects does not result in an immediate or long-term increase in circulating IGF-I levels. This lack of effect is probably due to the digestion of the IGF-I by intestinal digestive enzymes. These findings provide additional support for the safety profile of colostrum ingestion in populations that can benefit from the favorable effects of colostrum on gastrointestinal and respiratory health.
